# The *FpPPR1* Gene Encodes a Pentatricopeptide Repeat Protein That Is Essential for Asexual Development, Sporulation, and Pathogenesis in *Fusarium pseudograminearum*

**DOI:** 10.3389/fgene.2020.535622

**Published:** 2021-01-15

**Authors:** Limin Wang, Shunpei Xie, Yinshan Zhang, Ruijiao Kang, Mengjuan Zhang, Min Wang, Haiyang Li, Linlin Chen, Hongxia Yuan, Shengli Ding, Shen Liang, Honglian Li

**Affiliations:** ^1^Henan Agricultural University/Collaborative Innovation Center of Henan Grain Crops/National Key Laboratory of Wheat and Maize Crop Science, Zhengzhou, China; ^2^Xuchang Vocational Technical College, Xuchang, China; ^3^Horticulture Research Institute, Henan Academy of Agricultural Sciences, Zhengzhou, China

**Keywords:** *Fusarium pseudograminearum*, pentatricopeptide repeat, *FpPPR1*, mitochondrial, pathogenesis, wheat, aerial hyphae

## Abstract

*Fusarium* crown rot (FCR) and *Fusarium* head blight (FHB) are caused by *Fusarium pseudograminearum* and are newly emerging diseases of wheat in China. In this study, we characterized *FpPPR1*, a gene that encodes a protein with 12 pentatricopeptide repeat (PPR) motifs. The radial growth rate of the Δ*Fpppr1* deletion mutant was significantly slower than the wild type strain WZ-8A on potato dextrose agar plates and exhibited significantly smaller colonies with sector mutations. The aerial mycelium of the mutant was almost absent in culture tubes. The Δ*Fpppr1* mutant was able to produce spores, but spores of abnormal size and altered conidium septum shape were produced with a significant reduction in sporulation compared to wild type. Δ*Fpppr1* failed to cause disease on wheat coleoptiles and barley leaves using mycelia plugs or spore suspensions. The mutant phenotypes were successfully restored to the wild type levels in complemented strains. FpPpr1-GFP signals in spores and mycelia predominantly overlapped with Mito-tracker signals, which substantiated the mitochondria targeting signal prediction of FpPpr1. RNAseq revealed significant transcriptional changes in the Δ*Fpppr1* mutant with 1,367 genes down-regulated and 1,333 genes up-regulated. NAD-binding proteins, thioredoxin, 2Fe-2S iron-sulfur cluster binding domain proteins, and cytochrome P450 genes were significantly down-regulated in Δ*Fpppr1*, implying the dysfunction of mitochondria-mediated reductase redox stress in the mutant. The mating type idiomorphic alleles *MAT1-1-1, MAT1-1-2*, and *MAT1-1-3* in *F. pseudograminearum* were also down-regulated after deletion of *FpPPR1* and validated by real-time quantitative PCR. Additionally, 21 genes encoding putative heterokaryon incompatibility proteins were down-regulated. The yellow pigmentation of the mutant was correlated with reduced expression of *PKS12* cluster genes. Taken together, our findings on *FpPpr1* indicate that this PPR protein has multiple functions in fungal asexual development, regulation of heterokaryon formation, mating-type, and pathogenesis in *F. pseudograminearum*.

## Introduction

The winter wheat region in Huang Huai plain is the main wheat production area in China. Within this region, the Henan Province has the highest planting area and yield. A winter wheat-summer corn double cropping system has been the main manner of tillage in this region for many years. Wheat stubble on the soil surface or buried stubble are retained in this region to promote soil structure and nutrition (Zhang et al., 2007), however, this practice also allows for the accumulation of soil-borne fungal pathogens (Burgess et al., 1996). In recent years, *Fusarium* crown rot (FCR), mostly caused by soil-borne fungal pathogens *F. pseudograminearum, F. graminearum*, and *F. culmorum*, became an emerging disease in the Huang Huai winter region, leading to losses in yield (Zhou et al., 2014; Li et al., 2016). In the pathogen complex, *F. pseudograminearum* was predominant and had been detected in seven provinces (Zhou et al., 2019; Deng et al., 2020). FCR is a common disease of wheat and barley worldwide with typical symptoms of brown discoloration on the crown, leaf sheathes, lower stem tissues, and white heads (Smiley et al., 2005). Infected wheat by *F. pseudograminearum* might reduce kernel weight, numbers of kernels per head, grain weight, tiller height, and straw weight (Smiley et al., 2005). In Henan, the FCR caused 38.0 to 61.3% in yield losses from 2013 to 2016 (Xu et al., 2016). In Australia, nearly AU $80 million was lost annually due to FCR (Murray and Brennan, 2009). In the United States, FCR reduced winter wheat yields 9.5 to 35% in the commercial fields from 1993 to 2001 (Smiley et al., 2005).

*Fusarium* head blight (FHB), predominantly caused by *F. graminearum* in Huang Huai winter wheat region, was also detected by *F. pseudograminearum* in three provinces Henan, Hebei, and Shandong (Ji et al., 2015; Xu et al., 2015), which had led to epidemics in Australia (Burgess et al., 1987; Miedaner et al., 2010; Obanor et al., 2013; Kazan and Gardiner, 2017). In Henan, Li et al. (2017) isolated pathogens from 250 of the diseased wheat head samples in the fields and the detection rates for *F. gramraminum* and *F. pseudograminearum* were 83.3 and 55.6%, respectively. *F*. *pseudograminearum* can survive in stubble residues for up to three years, thereby increasing the amount of inoculum (Summerell and Burgess, 1988; Dodman and Wildermuth, 1989; Backhouse, 2014).

The screening of 88 local wheat cultivars-caused by *F. pseudograminearum* under artificial inoculation assay was conducted and showed that 88.64% (78/88) of the current varieties of wheat were susceptible or highly susceptible to FCR, while the remaining 10 cultivars exhibited moderate resistance at the adult stage (Yang et al., 2015). The mechanism of resistance to FCR remains obscure. Even some QTLs were identified for adult plant with partial resistance to crown rot by *F. pseudograminearum* in the bread wheat (Bovill et al., 2010), the low resolution of genetic mapping of major QTLs for the resistance of FCR is a major hurdle in wheat plant breeding (Liu et al., 2015).

*Fusarium pseudograminearum* is a hemibiotrophic fungus but the molecular mechanism involved in its pathogenesis has not been revealed. The entire genomes of *F. pseudograminearum* for the initial *MAT1-1* strain CS3096, the *MAT1-1* strain CS3270, along with the *MAT1-2* strain RGB5226 have been subsequently sequenced (Gardiner et al., 2012, 2016). In a comparative genomic analysis, Gardiner et al. (2012) identified the horizontal movement of the *FaAH1* gene from a bacterium and the *DLH1-AMD1* two-gene cluster. *FpAH1* and *FpDLH1* encode a putative amidohydrolase and a putative dienelactone hydrolase, respectively, contributing to the virulence of barley or wheat, while *FpAH1* appears to have a host-specific selection between barley and wheat. Wheat produces defense compounds such as the benzoxazolin class of phytoalexins 6-netgixt-benzoxazolin-2-one (MBOA) and benzoxazolin-2-one (BOA) (Villagrasa et al., 2006; Powell et al., 2017). The *Fusarium* Detoxification of Benzoxazolinone (FDB) gene cluster including *FDB1, FDB2*, and *FDB3* encoding a γ-lactamase, N-malonyltransferase, and GAL4-like Zn (II)_2_Cys_6_ transcription factor, respectively, are involved in BOA and MBOA detoxification in wheat (Gardiner et al., 2012; Kettle et al., 2015a,b, 2016).

The whole genome sequencing promotes virulence genes characterization in the pathogenesis. Wang L. M. et al. (2017) found that *FpPDE1* encodes a putative P-type ATPase that is required for full virulence on wheat and barley. Chen et al. (2018) reported that 26 genes out of 29 autophagy-related (ATGs) genes were induced with differential expression levels during early or late stages of wheat infection. FpLhs1, one of 14 Hsp70 proteins, regulated fungal development, asexual reproduction, and pathogenicity through the protein secretion pathway (Chen et al., 2019a). The basic leucine zipper (bZIP) FpAda1, was essential for vegetative growth, conidiation, and full virulence of wheat seedling hypocotyls and root growth. The expressions of *FpCdc42* and *FpCdc2* during cell cycle regulation were regulated by *FpAda1* (Chen et al., 2019b). The eleven genes encoding putative basic helix-loop-helix (bHLH) transcriptional regulators were differentially expressed during infection of wheat and three bHLH genes were induced during infection and contributed to virulence in wheat (Chen et al., 2019c).

To investigate secondary metabolism in this fungus, Kang et al. (2020) characterized a nonribosomal peptide gene, *FpNPS9*, where the deletion mutant showed normal colony morphology, growth rate, and conidiation, but infection was strongly restricted in wheat coleoptiles and heads, indicating an interaction with the endogenous defense systems of wheat. DON production was also down-regulated in the *FpNPS9* mutant. Zhang et al. (2020) investigated *FpDEP*1, a yeast DEP1 ortholog, which was found to be involved in Rpd3L complex regulation of pleiotropic functions, including vegetative growth, conidiation, pathogenicity, inhibition of host defense, reactive oxygen species (ROS) accumulation, and vacuole membrane biogenesis.

Pentatricopeptide repeat (PPR) proteins usually consist of arrays of a degenerate 35 amino-acid structural motif and are usually present in organelles of eukaryotic cells (Small and Peeters, 2000). There are 450 plus PPR members in higher land plants and fewer numbers in fungi and mammals (Lurin et al., 2004; Schmitz-Linneweber and Small, 2008). PPR proteins play important roles in growth and development in plants (Wang et al., 2018). Physiological effects on oxidative phosphorylation in defective PPR proteins can cause human diseases (Lightowlers and Chrzanowska-Lightowlers, 2008). The budding yeasts, *Saccharomyces cerevisiae* and *S. pombe*, and humans have only 15, 10, and 7 PPR proteins, respectively (Herbert et al., 2013). A PPR protein *ppr10* in *S. pombe* functions as a general translational activator, which is stabilized by *Mpa1*. The deletion of *ppr10* affects the accumulation of specific mitochondrial mRNAs (Wang Y. et al., 2017). Loss of *ppr3, ppr4, ppr6*, or *ppr10* leads to non-sexual flocculation and filamentous growth of cells (Su et al., 2017, 2018). In filamentous fungi, nine PPR genes were found in *Neurospora crassa* and four of them were functionally characterized, including one that encodes Cya-5 (Cytochrome *c* oxidase) which is involved in post-transcriptional processing. Both Cya-5 in *N. crassa* and PET309 in *Saccharomyces cerevisiae*, partial ortholog of PPR proteins, regulate *COXI* (Cytochrome c oxidase, aa3 type, subunit I) translation and stability. *COXI* expression is required for the synthesis of respiratory complex I and targeted RNA processes in mitochondria, but it is not clear whether the assembly factor CIA84 (complex I intermediate associated proteins is involved) is involved in RNA editing (Coffin et al., 1997; Moseler et al., 2012). The objective of this study was to identify the pentatricopeptide repeat (PPR) gene in *F. pseudograminearum* and to reveal the function of PPR gene in the filamentous phytopathogen.

## Materials and Methods

### Sequence Analysis

Sequences were analyzed using DNAstar software (Bioinformatics Software for Life Science-DNASTAR)^1^, BLASTn, or BLASTp (Nucleotide BLAST on National Center for Biotechnology Information)^2^, and local BLAST against the genome of *Fusarium oxysporum* f. sp. *lycopersici* 4287 and *F. pseudograminearum* CS3096^3^. The predicted protein PPR domains were analyzed by TPRpred in the MPI Bioinformatics Toolkit^4^. SMART analysis from the Pfam database with amino acid residues predicted the domains^5^. Alignment of amino acid sequences and phylogenetic analysis were performed using DNAMAN and MEGA6 software, respectively.

### Fungal Strains and Culture Conditions

The *F. pseudograminearum* strain used in this study was the local isolate WZ-8A reserved at Henan Agricultural University. Solid potato dextrose agar (PDA; 200 g peeled potato, 20 g dextrose, 15 g agar, and 1 L water), liquid Yeast Peptone Glucose media (YPG; 1% yeast extract, 2% peptone, and 2% dextrose), and sporulation media consisting of carboxymethyl cellulose liquid media (CMC; 2% solution of CMC) preparations as well as the culture conditions were adopted from a previous report (Wang L. M. et al., 2017).

### Gene Deletion and Complementation in *F. pseudograminearum*

The split-marker strategy was applied to delete the candidate gene following Catlett et al. (2003). The detailed approach for gene deletion in *F. pseudograminearum* was further described by Wang L. M. et al. (2017). Briefly, the amplification of upstream, downstream, and hygromycin fragment were amplified (The primers used in this study are listed in Supplementary Table 1). A mixture of upstream and downstream with hygromycin fusion fragments, D1H1 and D2H2, generated from recombinant fusion fragment D1-H-D2 (Figures 2A,B), were used for the transformation of protoplasts of *F. pesudograminearum* wild type WZ-8A. PCR screening of putative knockouts and further Southern blot analysis were prformed (Figure 2C).

To recover the defects deletion mutant, a fusion construct pFpPPR1-GFP with 1.6-kb native promoter was generated and transformed into protoplasts of the Δ*Fpppr1* deletion mutant. The GFP signal of the complemented transformants from conidia, germ tubes, and mycelia of candidates was checked with a Nikon fluorescent Eclipse inverted Ti-S microscope. The colony morphology, radial growth, conidiation, and pathogenicity were then evaluated.

### Biological Assays

A 5 mm plug was placed on a 9-cm Petri dish containing 15 mL of PDA medium. Colony diameter was measured after a 3 d incubation at 25°C for the *FpPPR1* deletion mutant, complemented strain, and the wild type WZ-8A. The measurement for each colony was recorded on a perpendicular plane, giving an average value. Each strain was subjected to three independent experiments with at least three plates each. The statistical analysis of our results was performed via Student’s *t*-test in Excel worksheet.

To test sporulation, 7 mm fungal mycelia blocks were put in a 250 mL flask containing 100 mL CMC media with shaking at 150 rpm for 5 d at 25°C. All tests had three replicates. Spore morphology and their germination were observed under a microscope. The spores at 1 × 10^5^/mL were dropped onto sterile glass slides. The slides were placed in a moist plastic container kept at 25°C. The spore germination rate was counted using at least 200 conidia with three repeats.

To determinate the defects of the mutants on reactive oxidative stress in *vivo*, the toleration to H_2_O_2_ was assayed on the solid medium plate containing 10 mM H_2_O_2_. For analysis of the response to different wall or membrane stresses, Congo red and sodium dodecyl sulfate (SDS) was supplemented in the culture medium containing 0.2 g/L Congo red or 0.05% (w/v) SDS. All the text of sensitivities to several stresses were carried out on 70-mm PDA plates at 25°C for 3 d. The perpendicular cross measurements method was applied to measure the colony diameter. The inhibition rate was calculated by formula inhibition rate = [(C−N)/(C−5)] × 100, where C is the colony diameter of the control and N is the colony diameter of the treatment (Chen et al., 2019).

### Pathogenicity Test

To determine pathogenicity, the seeds of the susceptible wheat cultivar Aikang58 were treated with 3% sodium hypochlorite solution for 3 min for sterilization, followed by three times washes with sterile water. After incubation in 10 cm diameter plates to accelerate germination, seedlings with shoots were placed on wet filter paper in a tray. The coleoptiles around 3 cm high were inoculated with 5-mm fungal block taken from 3 d old PDA cultures. The test had three replicates with 4-5 coleoptiles each. After 24 h of dark incubation at 25°C, fungal blocks were removed, and wheat plants were place in a greenhouse at 25°C (47% humidity) with a 16 h light/8 h dark photoperiod. After 3 d incubation, the plants were photographed.

Spore suspensions at 10^6^/mL or 5-mm fungal plugs from PDA plates after 3 d incubation at 25°C were inoculated on barley leaves for evaluation of the pathogenicity of *F. pseudograminearum* as described by Zhang et al. (2020). After treatment of 3% sodium hypochloride (NaOCl) solution for 3 min, seeds of barley cultivar Kenpimai 13 (generously provided by Baocang Ren, Research Associate, from Gansu Academy of Agricultural Sciences) was washed twice with sterile water and submerged in distilled water for 24 h in a beaker at 25°C. The imbibed seeds were placed into a plastic tray filled with a turfy soil mix (pH 5.5–7.0, humic acid ≥5.0%, organic substrate ≥25.0%, Shouguang Wode Agricultura Technology Co. LTD, Shandong, China). After planting, the tray was covered with plastic foil and put into a humid chamber (25°C, 16 h photoperiod, 47% humidity) for 5–7 d. The plastic foil was removed when the shoot reached 1 cm in height. Five to six barley seedlings (one formed leaf stage) were taken out of soil mix, laid horizontally in a new tray with the roots wrapped in a moist paper towel and leaves fixed on the surface of the tray by tape. Each treatment contained 5–6 leaves in a tray.

### Mitochondria Staining

Five to eight 7-mm fungal plugs from the leading edge of a 3-d-old colony of the complemented strain (cFpppr1) on PDA were transferred into a 250 mL flask containing 100 mL of CMC liquid media with shaking at 150 rpm at 25°C for 4–5 d. The conidia were harvested into a 50 mL microcentrifuge tube after filtering through one-layer sterile Miracloth. The conidia suspension was centrifuged at 2,500 *g* and supernatant was removed then resuspended in 1 mL of Phosphate buffered saline (PBS) and centrifuged again. After the second wash, 1 × 10^5^ conidia in a fresh tube were resuspended in prewarmed Mito-Tracker Red CMXRos M7512 at 37°C (MitoTracker mitochondrion-selective probes, Invitrogen) working solution (200 nM in PBS from the stock solution at −20°C, protected from light) for 10 min of staining following the kit instruction. The conidia were pelleted at 5,000 g for 3 min and resuspended in 50 μL of PBS for imaging with fluorescent GFPHQ green and TRITC red filters with a Nikon fluorescence Eclipse inverted Ti-S microscope.

### DNA Extraction and Southern Blot

Mycelia preparation for genomic DNA extraction and Southern blotting followed the previous method reported by Wang L. M. et al. (2017). The genomic DNA was extracted with the CTAB (2% CTAB (cetyl trimethyl ammonium bromide), 20 mM EDTA, 0.1 M Tris-HCl, and 1.4 M NaCl, plus 2% (v/v) 2-Mercaptoethanol added just before use) method (Lee et al., 1988). About 10 μg DNA of each strain was digested by *Xho* I, respectively, separated within a 0.8% agarose gel, blotted to Hybond N + membrane (GE Healthcare Life Sciences, United States), and detected following manufacturer’s instruction (Roche Diagnostics, United States) with *FpPPR1* fragment as probe 1 and the hygromycin resistance gene fragment as probe 2 labeled with Roche’s DIG High Prime DNA Labeling and Detection Kit II according to the manufacturer’s instructions.

### RNA-Seq Analysis

The wild type WZ-8A and mutant strains transferred to PDA plates from −80°C stock were incubated at 25°C for 3 d. A 1 cm square of the PDA culture block was cut and put into a blender (Weike ZW800D, Wenzhou Weike Biology Experiment Equipment Co., LTD) containing 100 mL of YPG liquid medium for 5 s of homogenization at 4,000 rpm/min. The homogenates were transferred into 250 mL flasks for mycelia growth rotating at 150 rpm/min at 25°C. After 3 d of growth, the mycelia were harvested by filtering through sterile Miracloth into a funnel (475855-R, EMD Millipore Corp)^6^ and rinsing mycelia twice with sterile distilled water.

For RNA-seq, three biological mycelia samples derived from wild type WZ-8A and the Δ*Fpppr1* deletion mutant were sent to Seqhealth Technology Co., LTD (Wuhan, China) for RNA extraction using TRIzol Reagent (Invitrogen, cat. NO 15596026)following the provided instructions. cDNA libraries were constructed using the KC-Digital^TM^ Stranded mRNA Library Prep Kit for Illumina^®^ (Catalog NO. DR08502, Wuhan Seqhealth Co., Ltd. China) following the manufacturer’s instruction. The library products around 200–500 bp in length were sequenced on a Novaseq 6000 sequencer (Illumina; PE150 model). The clean reads were mapped to the reference genome of *F. pseudograminearum* CS3096 (Gardiner et al., 2012)^7^ using the STAR software (version 2.5.3a) with default parameters. The mapped read counts were converted to RPKM (Reads per Kilobase per Million Reads). Genes differentially expressed between samples were identified using the edgeR package (version 3.12.1) (Robinson et al., 2010; McCarthy et al., 2012). The differentially expressed genes (DEGs) were defined via fold change (FC) in log2(FC) greater than 1.0 as calculated by the RPKM value of the same gene between mutant and wild type with the thresholds of both the p-value and the corrected *p*-value <0.05 to avoid false positives. Gene ontology (GO) analysis and Kyoto encyclopedia of genes and genomes (KEGG) enrichment analysis for differentially expressed genes were both implemented by KOBAS software (version: 2.1.1) with a p-value cutoff of 0.05 to assess statistically significant enrichment.

### Real-Time Quantitative PCR

For quantitative assays, the total RNA of wild type WZ-8A and Δ*Fpppr1* deletion mutant was extracted using the Trizol reagent (Ambion by Life Technologies 15596026) following the manufacturer’s instructions. The integrity of RNA quality was assessed by agarose gel electrophoresis and measured using an ultramicro spectrophotometer (Thermo). cDNA was synthesized according to the manual instructions of the PrimeScript^®^ RT reagent Kit (Perfect Real Time, TaKaRa Code: DRR047A). Briefly, the first strand cDNAs derived from 1 μg total RNA after removing the genomic DNA were synthesized in a 20 μL mix using an RT-PCR kit (PrimeScript RT reagent kit with gDNA eraser, code No. RR047A, TaKaRa) following the instructions. The 100 ng cDNA was used as the template in a 20 μL mix for qPCR using TB Green Premi Ex Taq II (Tli RNaseH Plus, Code No. RR820Q; TaKaRa). The cycling conditions were as follows: pre-denaturation at 95°C for 2 min followed by 40 cycles of denaturation at 95°C for 15s, annealing at 58°C for 15 s, extension at 68°C for 20 s, and a final stage at 95°C for 15 s to generate a melting curve. The RT-PCR was performed on Eppendorf realplex4 with software realplex Nr:6302 900 220-14. The quantitative results were calculated and analyzed using the 2^–ΔΔCt^ method with normalization to the CT value of an actin gene (Livak and Schmittgen, 2001). The gene candidates were selected to validate the RNA-seq results. The primers used in the test were listed in Table 1.

**TABLE 1:** Primers used in the quantitative real-time PCR.

Name	Sequence (5′-3′)	Description
FPSE_08050-RTF	AGGACAGAACATGGGGTCA	Quantitative real-time PCR primers for analysis of
FPSE_08050-RTR	GAGTACGAAACCAGATAGCATG	FPSE_08050 expression levels
FPSE_08176-RTF	CTATGAATTTGGAGGAACG	Quantitative real-time PCR primers for analysis of
FPSE_08176-RTR	TGCCAGTAGTCACGGATTTC	FPSE_08176 expression levels
FPSE_02237-RTF	TTCTTGAGGACCGTATTGG	Quantitative real-time PCR primers for analysis of
FPSE_02237-RTR	TATGACGAACTGGAGGAGC	FPSE_02237 expression levels
FPSE_00389-RTF	AAGCCAGTTATTATTGTCGG	Quantitative real-time PCR primers for analysis of
FPSE_00389-RTR	TCTTTGATTCCCATTCTGC	FPSE_00389 expression levels
FPSE_05514-RTF	CTTGGCGTAGACGTCATC	Quantitative real-time PCR primers for analysis of
FPSE_05514-RTR	GTGTCCAGGAGTGGGTTGC	FPSE_05514 expression levels
FPSE_05576-RTF	AGGCGACGGTCTAGGAGTC	Quantitative real-time PCR primers for analysis of
FPSE_05576-RTR	TTGGGAATGAGAACAAACAGC	FPSE_05576 expression levels
FPSE_08474-RTF	TCAGTGCCAGGAACTCTAC	Quantitative real-time PCR primers for analysis of
FPSE_08474-RTR	TGGACTTCATCTGTTACGC	FPSE_08474 expression levels
FPSE_05266-RTF	CCTTTCAACCCGCCTCATA	Quantitative real-time PCR primers for analysis of
FPSE_05266-RTR	CTGGCTTCAACAATAGTGTCC	FPSE_05266 expression levels
FPSE_06206-RTF	GTCATGTCCTGCTCTTTCACC	Quantitative real-time PCR primers for analysis of
FPSE_06206-RTR	GGTCAAGCCGAGGTACTC	FPSE_06206 expression levels
FPSE_07921-RTF	TGAGCTTCTACGAAACATC	Quantitative real-time PCR primers for analysis of
FPSE_07921-RTR	GCTTCACATTTGACCCAGT	FPSE_07921 expression levels
FPSE_04661-RTF	GCGTTGTTGATGCTTTGAG	Quantitative real-time PCR primers for analysis of
FPSE_04661-RTR	GACCTCCTTGGACACCTGA	FPSE_04661 expression levels
MAT1-1-1-RTF	CGAACCTACTACTTGAAGC	Quantitative real-time PCR primers for analysis of
MAT1-1-1-RTR	GTTACCAAAGTTGTCGTGA	MAT1-1-1 expression levels
MAT1-1-2-RTF	CACGCTTGAGGTCTTACGC	Quantitative real-time PCR primers for analysis of
MAT1-1-2-RTR	AGCAGATGGCAGATTTGGC	MAT1-1-2 expression levels
MAT1-1-3-RTF	TCACTCGTGATCAATCTACT	Quantitative real-time PCR primers for analysis of
MAT1-1-3-RTR	TTGTATCCAGGGTAAAGTC	MAT1-1-3 expression levels

## Results

### Identification of *FpPPR1* in *F. pseudograminearum*

From a screen of a T-DNA insertion library of *F. oxysporum* f. sp. *niveum* (*FON*) strain FON-11-06, we identified the FOXG_08514 gene in *Fusarium oxysporum* f. sp. *lycopersici* 4287 as a pentatricopetptide (PPR)-containing protein. The disrupted mutant exhibited a significantly reduced radial growth, less purple pigment on colony, and lost pathogenicity on watermelon seedlings. We continued to investigate its orthologue in *F. pseudograminearum* with significance in wheat production of Huanghuai winter regions.

**FIGURE 1:**
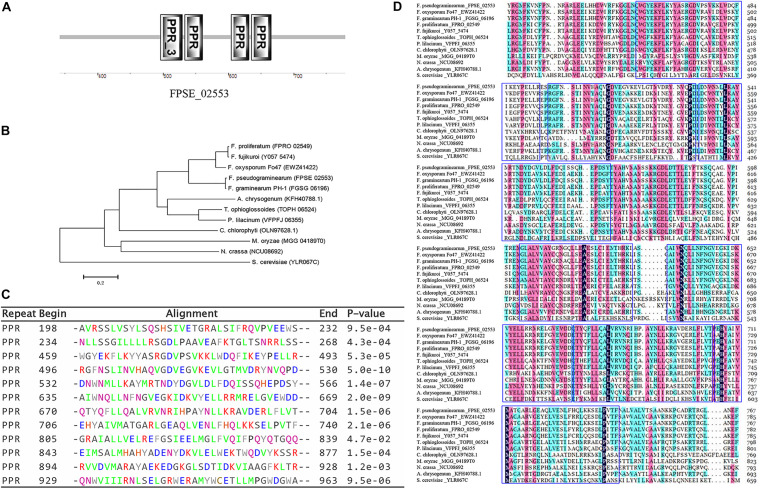
Identification of the PPR-like gene FPSE_02553. **(A)** SMART functional domain prediction. **(B)** Phylogenetic tree showing the evolutionary relationships of fungal *FpPPR1* proteins. Phylogenies were inferred using a PHYLIP-based program (MEGA 6) to create an unrooted phylogenetic tree. **(C)** The predicted protein was analyzed by TPRpred in the MPI Bioinformatics Toolkit (https://toolkit.tuebingen.mpg.de/#/tools/psiblast) and 12 PPR motifs were listed with their positions and *P*-value. **(D)** Sequence analysis was performed by DNAMAN software (partial alignment). The results show the conserved domains in proteins. *F. pseudograminearum* (FPSE_02553), *F. graminearum* PH-1 (FGSG_06196), *F. oxysporum* Fo47 (EWZ41422), *F. fujikuroi* coxI translation protein CYA5 (Y057_5474), *F. proliferatum* ET1 related to coxI translation protein CYA5 (FPRO_02549), *T. ophioglossoides* CBS 100239 Pentatricopeptide repeat-containing protein (TOPH_06524), *P. lilacinum* translation regulator (Cya5) (VFPFJ_06355), *C. chlorophyti* Pentatricopeptide repeat-containing protein, chloroplastic (OLN97628.1), *N. crassa* coxI translation protein CYA5 (cya-5) (NCU08692), *M. oryzae* (MGG_04189T0), *A. chrysogenum* ATCC 11550 Pentatricopeptide repeat-containing protein-like protein (KFH40788.1), and *S. cerevisiae* (YLR067C).

Local BLASTp against the *F. pseudograminearum* genome database using the FOXG_08514 amino acid sequence revealed the ortholog gene FPSE_02553 (Supplementary Document). We analyzed FPSE_02553 using the *F. pseudograminearum* genome database^8^ and transcriptome data for the local strain WZ-8A. The FPSE_02553 locus was on the third chromosome with a predicted cDNA of 3,624 bp and a predicted protein of 1,207 amino acid residues without introns. SMART analysis (Pfam) of the FPSE_02553 amino acid residues predicted PPR domains in 3 particular positions (Figure 1A)^9^. BLASTp analysis against UniProtKB determined the identities of the translated protein cya5 in *Fusarium langsethiae* and *F. oxysporum* from orthologs to cya5 at a range of 58.1 to 87.1% identities in *F. langsethiae, F. oxysporum, F. proliferatum, F. mangiferae*, and *Gibberella fujikuroi*, as well as the PPR proteins at 41.2 to 42.1% identities in *Tolypocladium ophioglossoide, T. paradoxum, Hypocrea jecorina, T. capitatum*, and *Escovopsis weberi*. The translated protein cya5 is also a PPR-containing protein. A phylogenetic tree was constructed based on the alignment of amino acids (Figure 1B). The 7 PPR-repeat conserved domains from BLAST results were listed. Further prediction through TPRpred in the MPI Bioinformatics Toolkit identified 12 putative PPR motifs consisting of 35 degenerate amino acids (Figures 1C,D)^10^. Therefore, we designated FPSE_02553 as an *F. pseudograminearum* PPR gene, *FpPPR1*.

### Knockout of *FpPPR1*

**FIGURE 2:**
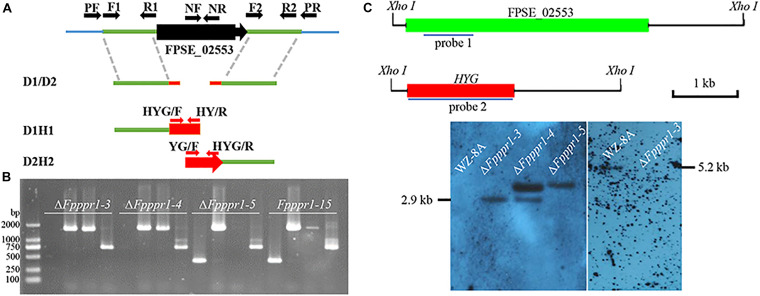
Knockout of *FpPPR1*. **(A)** Schematic diagram of the split marker PCR strategy. **(B)** Agarose gel electrophoresis of PCR products from transformants. M, DL = 2,000 bp. Δ*Fpppr1-3*, Δ*Fpppr1-4*, Δ*Fpppr1-5*, and Δ*Fpppr1-15* represent the transformants with hygromycin resistance. Each line was detected in order by the negative primer pair FpPPR1-NF/FpPPR1-NR, and the positive primer pairs FpPPR1-PF/H855R and H856F/FpPPR1-PR, and HYG-F/HYG-R, respectively, on a 1% agarose gel. **(C)** Southern blot hybridization analysis of the candidates. The schematic diagram on upper panel showed the cutting site of *Xho I* restriction enzyme in wild type (FPSE_02553) and mutant (*HYG*) genomes. The 1 kb drawing scale, probe 1 for detecting *HPH*, and probe 2 for detecting target gene were also listed. All genomic DNA from wild type WZ-8A, Δ*Fpppr1-3*, Δ*Fpppr1-4, and ΔFpppr1-5* were digested with *Xho* I. The lower panels showed the Southern blot results with probe 1 (right membrane) and probe 2 (left membrane), respectively. A 2.9-kb fragment was observed in Δ*Fpppr1-3* and Δ*Fpppr1-4*, but Δ*Fpppr1-4* is multiple copies. A 5.2-kb hybridized band was only detected in wild type WZ-8A, but not in Δ*Fpppr1-3* using probe 1 on downstream fragment of *FpPPR1*.

To determine the function of *FpPPR1* in *F. pseudograminearum*, we used a split-marker strategy to obtain hygromycin-resistant knockout transformants. After PCR screening with negative and positive primer pairs, three candidates were used for further Southern blot analysis. When hybridized with the hygromycin gene as a probe, Δ*Fpppr1-3* and Δ*Fpppr1-4* had a 2.9-kb band, but Δ*Fpppr1-4* with one extra insertion event. Δ*Fpppr1-3* strain was no band detected (wild type had a band of 5.2 kb) when hybridized with the *FpPPR1* fragment as a probe, which suggested that Δ*Fpppr1-3* was the clean knockout mutant (Figure 2C). The Δ*Fpppr1-3* strain would be used as the Δ*Fpppr1* mutant in the following assays.

The band of 2.9-kb *Xho* I indicates the target gene replacement, while one extra band suggests that the single copy insertion event occurred after the homologue replacement from two fragments of hygromycin gene. At the meantime, the random insertion of the fused hygromycin gene with function in the genome, might potentially display a side effects besides the target gene deletion. The Δ*Fpppr1-5* line was just an ectopic insertion strain without the target gene replacement events (Figure 2C), where the fused fragment including hygromycin gene was inserted in the genome randomly.

### Δ*Fpppr1* Exhibits Deficient Vegetative Growth, Weaken Conidiation, and Altered Conidia Morphology

**FIGURE 3:**
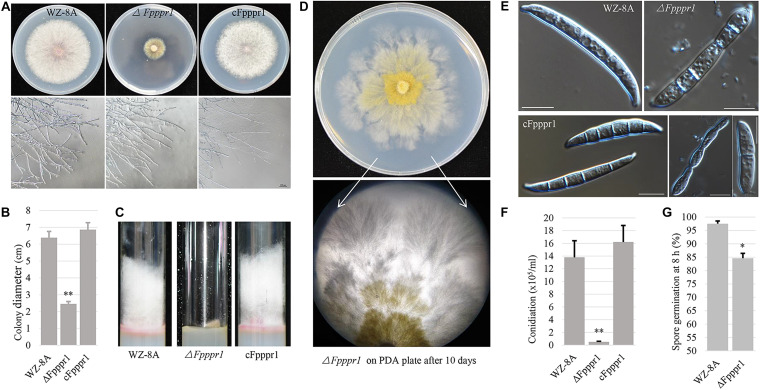
Characteristics of wild type (WZ-8A), Δ*Fpppr1*, and cFpppr1. **(A)** Colony phenotype with and mycelial branching patterns on PDA 3 d after inoculation; **(B)** Colony diameter measurement on PDA 3dpi (day post inoculation). **(C)** Comparison of aerial hyphal formation in culture tube. **(D)** Sectoring of colonies on a PDA plate. **(E)** Spore morphology. **(F)** Conidia produced in CMC media with inoculation of fungal plugs for 4–5 d with shaking at 25°C. **(G)** Germination of spore suspensions on glass slides at room temperature with quantification of spore germination after 8 h. The asterisk indicates significance (^∗^*p* < 0.05, ^∗∗^*p* < 0.01).

Colonies of the Δ*Fpppr1* mutant on PDA plates were significantly smaller (*p* < 0.01) than those of WZ-8A and the complemented strain with almost no aerial hyphae that were common in the wild type strain (Figures 3A–C). Δ*Fpppr1* hyphae exhibited yellow color with several round sector branching points when grown to almost reach the edge of Petri dish for 10 d compared to the wild type WZ-8A and the complemented strain (Figure 3D). Δ*Fpppr1* spore morphology was abnormal with constriction in the septum and large portions of spores only had one septum and the germination rate was significantly reduced (*p* < 0.05) compared to WZ-8A and the complemented strain after 8 h in a drop of sterile water at 25°C (Figures 3E,G). Spore production in CMC media was reduced by 28-fold after a 4-d incubation compared with wild type (Figure 3F).

### The Δ*Fpppr1* Functional Complementation and the *FpPpr1* Protein Localized to the Mitochondria

To functionally rescue the mutant, an FpPpr1-GFP fusion construct driven by a native promoter was generated and sequenced. When this GFP complementation construct was introduced to the protoplast of Δ*Fpppr1-3*, the FpPpr1-GFP transformants grew on PDA plates containing G418 antibiotics for selection of transformants resistant to neomycin. The phenotypic defects of the Δ*Fpppr1-3* mutant were successfully restored in verified complementation lines.

**FIGURE 4:**
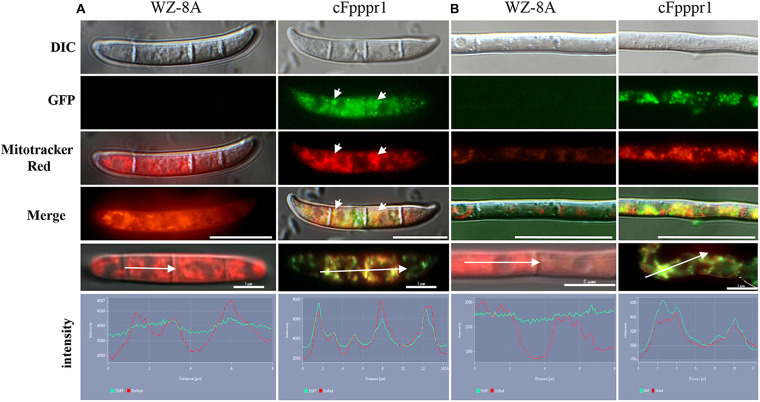
The localization of FpPpr1-GFP in *F. pseudograminearum.* Mito-tracker Red CMXRos M7512 staining on spores **(A)** and hyphae **(B)**. The spores were harvested from CMC medium into 50 mL microcentrifuge tubes and filtered through single-layer sterile Miracloth, centrifuged at 2,500 *g*, and resuspended with 1 mL of PBS and centrifuged again. The fresh spores were resuspended in prewarmed Mito-Tracker Red CMXRos M7512 (200 nM in PBS from the stock solution at –20°C protected from light) at 1 × 10^5^ spores/mL after two washes and incubated at 37°C (MitoTracker mitochondrion-selective probes, Invitrogen) for 10 min following the kit instructions. The conidia were pelleted at 5,000 g for 3 min and resuspended in 50 μL of PBS for imaging via fluorescent GFPHQ green and TRITC red filters with a Nikon fluorescence Eclipse inverted Ti-S microscopy. Red signal indicates mitochondrial localization. Green signal indicates the expression of the C-terminal GFP fusion protein FpPpr1-GFP. Yellow signal indicates co-localization of the green and red fluorescence signals. Scale bars indicate 20 μm. White arrows showed the obvious co-location site and direction of fluorescence intensity. Scale bars indicate 5 μm.

In order to determine the localization of the *FpPpr1* protein, the GFP complementation strain with a C-terminal GFP fusion to *FpPpr1* was examined under a Nikon microscope. GFP fluorescence was observed in an intracellular granule-like structure within spores and hyphae. As the *FpPPR1* proteins were predicted to be localized in the mitochondria by Wolf PSORT analysis^11^ and a mitochondria localization signal was found in amino acids 1 to 30 of FpPpr1^12^, further Mito-tracker Red CMXRos M7512 staining was performed. Some of the GFP signal overlapped with the Mito-tracker red signal, implying that *FpPPR1* was localized in the mitochondria, some signals appearing to localize in the cytosol (Figures 4A,B).

### *FpPPR1* Is Involved in Response to Cell Membrane and Cell Wall Stresses

**FIGURE 5:**
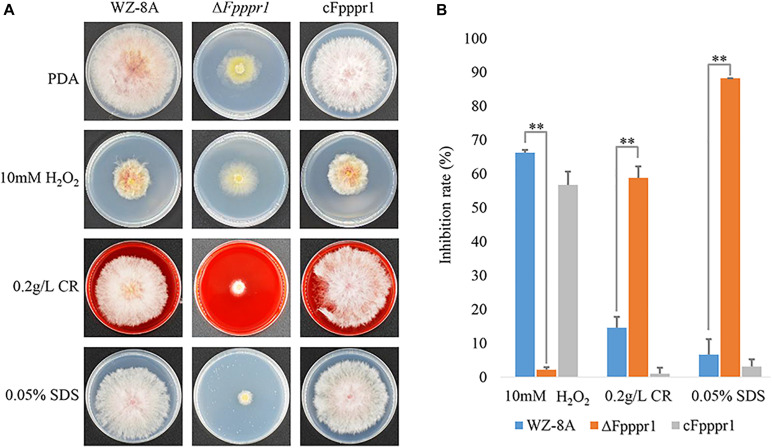
Evaluation of responses to various stresses H_2_O_2_, Congo red, and SDS. **(A)** Colonies of wild type WZ-8A, Δ*Fpppr1* mutant, and complemented strain cFpppr1 on media with 10 mM H_2_O_2_, 0.2 g/L Congo red, or 0.05% (w/v) SDS. **(B)** Mycelial growth inhibition compared with non-treated controls following incubation for 3 days on PDA containing 10 mM H_2_O_2_, 0.2 g/L Congo red, or 0.05% (w/v) SDS (^∗∗^*p* < 0.01).

On PDA containing 10 mM H_2_O_2_, Δ*Fpppr1* mutant growth had no obvious defect compared to WZ-8A and the complemented strain cFpppr1 (Figure 5A). In presence of 0.2 g/L Congo red and 0.05% SDS to stress the cell wall and cell membrane, respectively, colony growth was more stunted in Δ*Fpppr1* mutant compared to the other lines (Figure 5A). The calculated values showed a significantly increased inhibition rate (*p* < 0.01) in the Δ*Fpppr1* mutant under 0.2 g/L Congo red and 0.05% SDS, but the inhibition rate under 10 mM H_2_O_2_ was significantly lower (*p* < 0.01) than that of WZ-8A and the complemented strain cFpppr1 (Figure 5B).

### *FpPPR1* Is Essential for Infecting Barley Leaves and Wheat Coleoptiles

**FIGURE 6:**
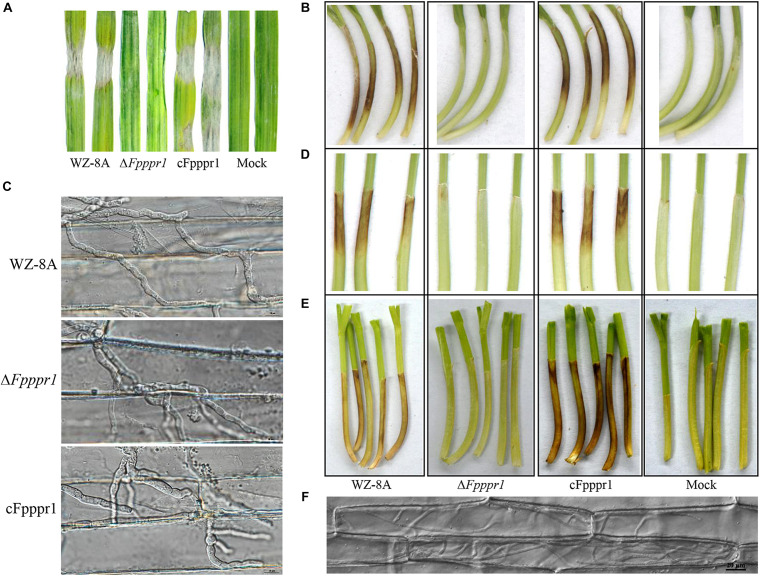
Pathogenicity test assay using WZ-8A, Δ*Fpppr1*, and cFpppr1. **(A)** Barley leaves were inoculated with fungal mycelia plugs (5 mm diameter) or PDA plugs without mycelia as a mock. **(B)** Wheat coleoptiles with inoculation of fungal or PDA plugs for 3 d. The plugs were removed after 24 h. **(C)** Histopathological images of infectious hyphae in the abaxial epidermis of wheat coleoptiles 3 dpi. Scale bars indicate 10 μm. **(D)** Wheat coleoptiles were inoculated with spore suspension and sterile water as mock and photographed at 8 dpi and **(E)** at 16 dpi. **(F)** Histopathological images of infectious hyphae in the abaxial epidermis of wheat coleoptiles 14 dpi. The images were taken under Nikon Eclipse Ti-S microscopy in DIC (differential interference contrast) model. Scale bars indicate 20 μm.

To characterize the potential defects of host infection, fungal plugs of 3-day-old *F. pseudograminearum* were placed onto intact barley leaves attached to the bottom of the growth chamber facing up. WZ-8A and cFpppr1 caused large necrotic lesions in size on intact barley leaves by 3 d post-inoculation (dpi) after the fungal plugs were removed. In contrast, no lesions were found on the Δ*Fpppr1* mutant inoculated on intact barley leaves (Figure 6A). The fungal plugs were also put on intact wheat coleoptiles fixed horizontally on the bottom of a growth chamber. Brown or dark brown lesions were observed 3 d in coleoptiles inoculated with WZ-8A and complemented strains, while no discoloration of coleoptiles was observed from the Δ*Fpppr1* mutant (Figure 6B). The transparent inner epidermis was stripped off and observed under a microscope. The infection mycelia from the mutant could be observed and showed no obvious morphological difference compared to wild type and cFpppr1 strains (Figure 6C). However, elongated mutant spore inoculation on coleoptiles did not cause lesions after 8 d (Figure 6D) or even 16 d (Figure 6E). Observing the inner epidermis of wheat coleoptile inoculated with 14 d, it was found that there were a lot of mycelium in it, but the wheat coleoptile did not show obviously dark brown or necroses symptom (Figure 6F and Supplementary Figure 3).

### Genes Regulated by *FpPPR1* Are Involved in Oxidoreductive Reactions, Mating, and Secondary Metabolism

**FIGURE 7:**
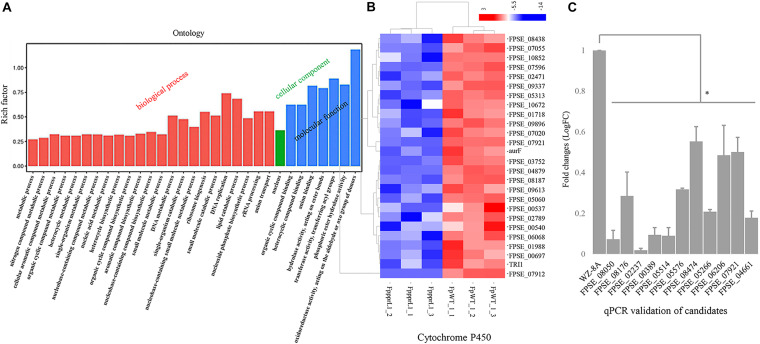
Expression profiles of specific genes in the Δ*Fpppr1* mutant compared to wild type. **(A)** Expression profiles of specific genes for Δ*Fpppr1* and wild type strain WZ-8A in three biological replicates. The deferentially expressed genes (DEGs) were in log2 (fold change, FC) greater than 1.0 with a threshold at p-value and corrected *p*-value <0.05 (False positive). **(B)** Heatmap for cytochrome P450 cluster. **(C)** Real time qRT-PCR for differentially down-regulated genes encoding oxidoreductases. First-strand cDNAs were synthesized using an RT-PCR kit as templates for qRT-PCR. The quantitative results are calculated and analyzed using the 2^–ΔΔCt^ method (^∗^*p* < 0.05). FppprL1_1, FppprL1_2 and FppprL1_3 represent the sample names of the Δ*Fpppr1* mutant strain for RNA-seq. FpWT_1_1, FpWT_1_2, and FpWT_1_3 represent the samples names of wild type strain WZ-8A for RNA-seq.

To explore the regulatory role of *FpPPR1* at a genome-wide scale, transcriptomic profiles were generated by the RNA-seq from wild type and the ΔFpppr1 mutant (RNA-seq data, submission No. SUB7037132)^13^. A total of 1,367 genes were differentially down-regulated, while 1,333 genes were differentially up-regulated (all the expression analysis at *p* < 0.05 and FDR < 0.05). Since FpPpr1-GFP largely localized to mitochondria, we noticed that down-regulated genes were enriched for molecular functions relevant to oxidoreductase activity during GO clustering analysis (Figure 7A). Twenty-six cytochrome P450 genes were significantly down-regulated (9 in E-class group I, 7 in E-class group IV, and 10 in drug metabolism) (Figure 7B). We verified 10 genes encoding FAD or NAD-binding domain-containing oxidoreductases (Supplementary Table 2 with description) and FPSE_07921, encoding a putative E-class group I cytochrome P450, had significantly reduced expression levels after deletion of *FpPPR1* using qRT-PCR (Table 1 with primers; Figure 7C). Three genes, FPSE_11993, FPSE_12025, and FPSE_00773, encoding the 2Fe-2S iron-sulfur cluster binding domain proteins were also significantly down-regulated. Expression of FPSE_01622, encoding a thioredoxin TRX group I ortholog of TRX1 in *Magnaporthe oryzae*, was significantly reduced as well.

**FIGURE 8:**
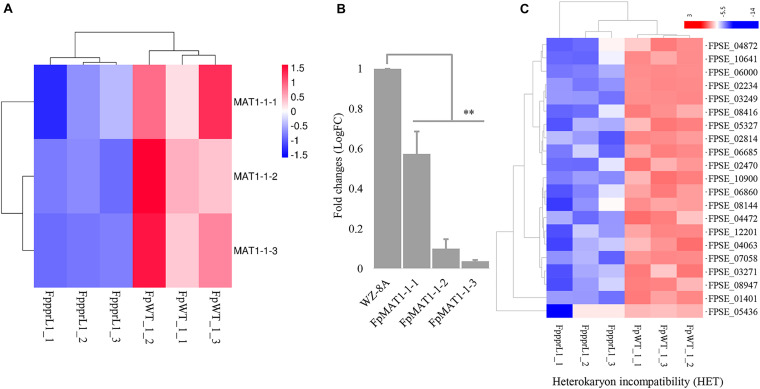
The gene expression profiles on the mating type locus *MAT1* and heterokaryon incompatibility. **(A)** Heatmap of the *MAT1-1* idiomorphs *MAT1-1-1, MAT1-1-2*, and *MAT1-1-3* with reduced expression levels in Δ*Fpppr1* mutant, data derived from RNA-seq and qRT-PCR confirmed **(B)**. **(C)** Heatmap for genes encoding heterokaryon incompatibility proteins (HET) (^∗∗^*p* < 0.01).

The three *MAT1-1* mating-type alleles, *FpMAT1-1-1, FpMAT1-1-2*, and *FpMAT1-1-3*, showed significantly down-regulated expression levels in mutant Δ*Fpppr1* (Figure 8A), which was also confirmed by qRT-PCR (Figure 8B). There were 21 gene-encoded putative heterokaryon incompatibility proteins (HET) with significantly down-regulated expression levels in Δ*Fpppr1* (Figure 8C).

**FIGURE 9:**
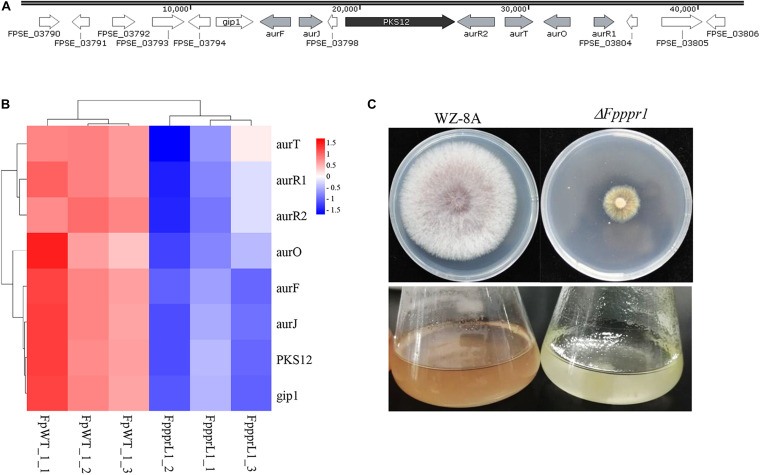
The *PKS12* cluster is responsible for aurofusarin biosynthesis. **(A)** Retrieved genomic sequences and mapping of the *PKS12* cluster. The arrows indicate ORFs and transcriptional directions, *AUR* genes are gray, *PKS12* is black, and other genes are white. **(B)** Heatmap of the *PKS12* cluster with reduced expression levels in Δ*Fpppr1* mutant from RNA-seq data. **(C)** The comparison of pigmentation from wild type WZ-8A and the Δ*Fpppr1* mutant on PDA plates and CMC liquid media.

The *PKS12* (polyketide synthase 12) gene cluster, including *PKS12, aurF, aurJ, aurO, aurR1, aurR2, aurT*, and *gip1* in Figure 9A showed significantly down-regulated expression levels (Figure 9B), which was responsible for aurofusarin pigmentation the Δ*Fpppr1* mutant exhibited yellow coloration on PDA plates and in liquid CMC media (still sticky after several days incubation) (Figure 9C). Besides *PKS12, PKS2, PKS6, PKS10*, and *PKS14* also exhibited significantly lower expression levels in the Δ*Fpppr1* mutant.

## Discussion

In this study, we first characterized a putative *F. pseudograminearum* PPR gene *FpPPR1* in a filamentous phytopathogen, which encodes a protein containing 12 repeats of a degenerate 35-amino-acid motif. FpPpr1 is involved in multiple biological processes including aerial hyphae development, vegetative growth, asexual sporulation, mating type gene expression, secondary metabolism and pathogenicity. Although more efforts are needed to validate these regulation pathways indicated by transcriptome data, we demonstrated that the FpPpr1 are necessary for oxidoreductive system in mitochondria. We identified a disrupted gene with PPR motifs in *F. oxysporum* f. sp. *niveum* by ATMT (*Agrobacterium tumefaciens*-mediated transformation) displayed multiple defects including a reduced growth rate with white colony coloration, short and highly dense aerial hyphae, and reduced virulence on watermelon seedlings (Supplemental Document). The *FpPPR1* ortholog gene in *F. pseudograminearum* showed similar functions. Through DNAMAN aliment FpPpr1 shares 83.7% identity with putative *cya*-*5* orthologs in *Fusarium* spp. The *cya-5* pentatricopeptide repeat (PPR) protein in *Neurospora crassa* (Coffin et al., 1997), ortholog of yeast Pet309 PPR protein, which shared about 20% amino acid identity with FpPpr1, was required for post-transcriptional modification of COXI (cytochrome c oxidase subunit I), and the mutant was deficient in accumulation of mitochondria COX1 pre-RNA and translation of COX1 RNAs in yeast (Manthey et al., 2010). The spore germination of *N. crassa* was dependent upon the function of the cytochrome-mediated electron transport pathway within mitochondria. Transcriptomic analysis by RNA-seq from the Δ*Fpppr1* mutant and wild type revealed that large numbers of genes displayed differential expression after *FpPPR1* deletion. Among the genes with differentially down-regulated expression levels, clustering analysis mapped the highest proportion of such genes into the gene products with oxidoreductive activity.

FpPpr1 contains a mitochondria localization signal and was found to be predominantly localized to said organelle. Previous studies have characterized the role of nuclear-encoded PPRs and similarly found mitochondria and plastid localization, with potential functions in targeting RNAs for RNA metabolism (Shikanai, 2006; Zehrmann et al., 2011; Giegã, 2013; Li and Jiang, 2018; Wang et al., 2018; Yan et al., 2018). However, FpPpr1 has not yet been shown to target RNAs, but assessing FpPpr1 binding of RNA in *F. pseudograminearum* is worth exploration. The PPR gene family in plants is involved in the response to environmental stimuli including biotic and abiotic stresses such as fungal infection, salicylic acid (SA), methyl jasmonate (MeJA), mechanical wounding, and cold and salinity stress (Kobayashi et al., 2007; Tang et al., 2010; Xing et al., 2018).

The Δ*Fpppr1* mutant exhibited reduced radial growth and colony sectoring. Li et al. (2008) reported that high oxidative stress, decreased membrane integrity, and DNA glycosylation in the mitochondria of mycelia contribute to the sectorization of colonies on PDA plate in the entomopathogenic soil fungus *Metarhizium anisopliae.* Mutation of the Mgv1 ortholog lt2 from *Cryphonectria parasitica* led to abnormal cell wall integrity and sectorization (So et al., 2017). Overexpression of the *GIP2* transcription factor (also called aurR1) in aurofusarin biosynthesis caused sector formation and high pigmentation inhibited normal early vegetative growth in *Gibberella zeae* (Kim et al., 2006). The impaired aurofusarin biosynthesis likely led to the accumulation of the intermediate rubrofusarin, causing the yellow coloration on PDA plates. Our transcriptome data revealed similar defects in oxidative stress management and transmembrane components, and down-regulated aurofusarin biosynthesis in the mitochondria of mycelia, while the effects on membrane potential need to be measured to support links to previous findings.

The major pathogens of *Fusarium* crown rot, *F. pseudograminearum, F. graminearum*, and *F culmorum*, all produce the red pigment aurofusarin, a dimeric polyketide naphthoquinone (Medentsev and Akimenko, 1998; Malz et al., 2005; Cambaza, 2018). A type I PKS (polyketide synthase) gene cluster, *PKS12*, is responsible for aurofusarin synthesis (Frandsen et al., 2006). There are 14 putative PKSs in *F. pseudograminearum* and 5 of them (*PKS2, PKS6, PKS10, PKS12*, and *PKS14*) were down-regulated in the Δ*Fpppr1* mutant. Malz et al. (2005) identified the *PKS12* gene cluster including 10 genes required for the biosynthesis of aurofusarin in *F. pseudograminearum* through *Agrobacterium turmefaciens*-mediated mutagenesis via T-DNA insertion. Our results suggest that FpPpr1 is at least involved in the regulation of the *PKS12* gene cluster in *F. pseudograminearum*. In *F. graminearum*, histone H3 lysine 4 methylation regulates the biosynthesis of aurofusarin (Liu et al., 2015; Cambaza, 2018). The *PKS12* gene seems not to affect pathogenicity in *F. pseudograminearum* (Malz et al., 2005), while more zearalenone (ZEA) was produced in the Δ*pks12* mutant. Aurofusarin negatively regulates zearalenone biosynthesis (Malz et al., 2005). *F. pseudograminearum* produces DON and ZEA as well (Blaney and Dodman, 2002), where DON plays an important role in virulence during *Fusarium* crown rot (Powell et al., 2017). Whether the attenuated virulence of the mutant has potential association with the mycotoxin reduction, DON and ZEA production in the Δ*Fpppr1* mutant needs to be measured.

*F. pseudograminearum*, also known as *Gibberella coronicola* (teleomorph), is a heterothallic fungus and the sexual process is controlled by opposite and distinct MAT idiomorphs, which include *MAT1-1* (*MAT1-1-1, MAT1-1-2*, and *MAT1-1-3* isoforms) and *MAT1-2* (*MAT1-2-1* and *MAT1-2-3* isoforms) loci in different strain, respectively (Gardiner et al., 2016). In the Δ*Fpppr1* mutant background, the expression levels of three mating type alleles in *MAT1-1* locus were significantly dropped off suggesting as a regulator in the sexual reproduction of *F. pseudograminearum*. Although there were still vary effects among the alleles. The smaller effect of FpPpr1 to *MAT1-1-1* was showed indicating that another factor exists through affecting this PPR gene. Whether the *MAT1-2* idiomorphs are regulated by *FpPPR1* still needs to be characterized. PPRs in plants have functions in restoring cytosolic male sterile (CMS) through regulating the genes related to CMS (Schmitz-Linneweber and Small, 2008; Lydiane et al., 2016). In *Ustilago maydis*, a dimorphic switch between yeast-like and filamentous growth is controlled by the mating-type loci (Wösten et al., 1996). Whether *FpPPR1* on regulation of the mating type loci in *F. pseudograminearum* goes through aerial hyphae development still needs to be characterized. Sexual recognition is controlled by the mating-type locus and the vegetative recognition is controlled by heterokaryon incompatibility systems in filamentous ascomycetes (Saupe, 2000). In transcriptome data of Δ*Fpppr1* background, a group of genes encoding heterokaryon incompatibility proteins (HET) in mutant background were significantly reduced in expression level. The FpPpr1 upregulated the *HET* expression level might contribute the inhibition of heterokaryon formation in opposite MAT strains. In contrast, mutation of *FpPPPR1* might result the heterokaryon formation, which might trigger parasexual process to be the potential genetic variation sources. The MAP kinase *MGV1* is essential for female fertility, heterokaryon formation, and plant infection in *F. graminearum* (Hou et al., 2002). The mgv1 mutant was female sterile and its ortholog (FPSE_12362) was significantly down-regulated in the Δ*Fpppr1* mutant. The MAP kinase function is usually conserved in filamentous fungi. Therefore, FpPpr1 might potentially determine self/nonself recognition in vegetative, thereby contributing to genetic variation in *F. pseudograminearum*. Although the perithecia was not difficult to find in the field, it is still not such population like *F. graminearum*.

Aerial hyphae (or mycelia on substrate surface) are essential for asexual and sexual development to produce propagules and initiate opposite sexual recognition. The Δ*Fpppr1* mutant was defective in aerial hyphae formation. Sporulation was significantly reduced in the Δ*Fpppr1* mutant, which indicates the aerial hyphae were still available in CMC liquid media. Whether or not sexual crossing is lost needs to be confirmed. Hydrophobins are thought to reduce surface tension for hyphae growing into the air in filamentous bacteria and fungi (Wessels et al., 1991; Wösten and Willey, 2000; Bayry et al., 2012; Riquelme et al., 2018). In filamentous fungi, most autophagy genes, such as *TrATG5, ATG15, BcATG8, AoATG1, AoATG26, MoATG14*, and *MoATG24*, regulate aerial hyphae development and sporulation or conidia germination (Liu et al., 2011, 2017; He et al., 2013; Yanagisawa et al., 2013; Kikuma et al., 2017; Ren et al., 2018). Deletion of the related gene blocks significantly reduces aerial hyphae formation and sporulation (Kikuma et al., 2007; Lv et al., 2017; Ren et al., 2018; Liu et al., 2019). Most of the ATG genes are critical to selective mitochondrial autophagy for mitochondrial stasis (Marinkovic and Novak, 2015). The RNA-seq results from the Δ*Fpppr1* mutant did not reveal differentially expressed genes encoding hydrophobic proteins and *ATG* orthologs, which might indicate a novel mechanism through which *FpPPR1* regulates mating-type genes in *F. pseudograminearum* (Lugones et al., 2004; Elliot and Talbot, 2005). It is interesting that the key component, MGV1, in *F. graminearum* in the cell wall integrity (CWI) pathway Bck1-Mkk1-Slt2-Rlm1 was detected with a down-regulated expression level in Δ*Fpppr1* (Hou et al., 2002; Levin, 2005, 2011). The MAP kinase *mgv1* mutant produced fewer and shorter aerial hyphae with less pigmentation. Sensitivity to Congo red and SDS also suggested defects in the cell membrane and cell wall. The orthologs of AtSlt2 in *Alternaria alternata* (Yago et al., 2011), Mpka in *Aspergillus nidulans* (Bussink and Osmani, 1999), Mgslt2 in *Mycosphaerella graminicola* (Mehrabi et al., 2006), Bmp3 in *Botrytis cinerea* (Rui and Hahn, 2007), FoSlt in *Fusarium oxysporum* f. sp. *cubense* (Ding et al., 2015), and CpSlt2 in *Cryphonectria parasitica* (So et al., 2017), and Mps1 in *M. oryzae* (Xu et al., 1998) shared similar phenotypes in mutants with fewer and shorter aerial hyphae of the colonies. Likewise, the deletion mutant of the ortholog *MgSlt2* in *Mycosphaerella graminicola* also did not produce aerial mycelia even after prolonged growth on PDA (Mehrabi et al., 2006). The *FpPPR1* gene in different filamentous phytopathogens might have a conserved function in regulating aerial hyphae development through the CMI MAPK pathway. The Δ*Fpppr1* mutant exhibited no pathogenicity when inoculated as a fungal mycelial plug and spore suspension onto plant tissue. Interestingly, if the fungal agar was not removed from coleoptiles, the Δ*Fpppr1* mutant could still cause lesions indicating the importance of aerial hyphae for infectious structure formation. *F. pseudograminearum* can form a foot-like appressorium and invade the neighboring cell for extension into host tissue. We speculate that the fewer, shortened aerial hyphae on the agar are capable of forming appressoria to penetrate the host epidermis. Along these lines, our histopathological images showed normal growth of infectious hyphae in the wheat coleoptile cells. This is similar to what was observed for *MgSlt2* mutants in *M. graminicola* which showed reduced virulence with no branching infectious hyphae (Mehrabi et al., 2006).

In summary, our findings uncovered new insight into pentatricopeptide repeat proteins in the filamentous phytopathogens *F. pseudograminearum* and *F. oxysporum* f. sp. *niveum*. We discovered multiple PPR functions related to growth, aerial hyphae development, sporulation, and regulation of mating type gene *MAT1-1*. To fully understand the roles of PPR proteins and to determine whether RNA processing also occurs in mitochondria in *F. pseudograminearum*, the seven remaining PPR proteins in *F. pseudograminearum* need to be characterized.

## Data Availability Statement

The original contributions presented in the study are publicly available. This data can be found here: https://www.ncbi.nlm.nih.gov/Traces/study/?acc=PRJNA674906.

## Author Contributions

LW, YZ, SX, and SL wrote the manuscript. HLL, HYL, and SD revised the manuscript. LW, YZ, and SX performed the majority of the experiments in *F. pseudograminearum* with participation from RK, MZ, MW, LC, and HY. SL performed all the experiments in *F. oxysporum* f. sp. *niveum*. All authors contributed to the article and approved the submitted version.

## Conflict of Interest

The authors declare that the research was conducted in the absence of any commercial or financial relationships that could be construed as a potential conflict of interest.

## Footnotes

1 www.dnastar.com

2 https://www.ncbi.nlm.nih.gov/

3 http://fungi.ensembl.org/

4 https://toolkit.tuebingen.mpg.de/#/tools/psiblast

5 http://smart.embl-heidelberg.de

6 www.millipore.com

7 https://mycocosm.jgi.doe.gov/Fusps1/Fusps1.home.html

8 http://fungi.ensembl.org/Fusarium_pseudograminearum/Info/Index

9 http://smart.embl-heidelberg.de

10 https://toolkit.tuebingen.mpg.de/#/toolspsiblast

11 https://www.genscript.com/wolf-psort.html?src=leftbar

12 http://ipsort.hgc.jp/predict.cg

13 https://www.ncbi.nlm.nih.gov/Traces/study/?acc=PRJNA674906

## Supplementary Material

Supplementary Figure 1 Vectors used for *Agrobacterium tumefaciens*-mediated transformation of *Fusarium oxysporum* f. sp. *niveum* (*FON*) strain FON-11-06, the special structure of the T-DNA region in pATMT1-EGFP and pATMT1 vectors for high-efficiency nested inverse (HENI)-PCR, and a schematic diagram for identifying the sequences flanking the T-DNA region of interest. **(A)** pCAMBIA1300 vector, **(B1)** pATMT1 vector, and **(B2)** pATMT1-EGFP vector; Panels **(B1)** and **(B2)** were suitable for HENI-PCR amplification of unknown sequences flanking both borders of T-DNA in transgenic *FON* mutants; **(C1–C3)** schematic diagram for identifying sequences flanking the T-DNA region of interest; **(D)** special T-DNA structure for HENI-PCR in pATMT1-EGFP vector and primer design. **(E)** Numbers 1 to 12 in panel **(C)** correspond to 12 primers, namely ILP, ILP1, IRP1, IRP2, ILP4, ILP3, ILP6, ILP5, IRP3, IRP4, ILP8, and ILP7, respectively.

Supplementary Figure 2 Morphology and pathogenicity assay for the mutant W1005D in *FON* strain FON-11-06. **(A)** The wild type and mutant cultures on PDA plates after 7d at room temperature. **(B)** The sprouting watermelon seeds were inoculated directly with 0.5 mL of the spore suspension at 1 × 10^5^/mL in the tray cells. The sterile water was dropped onto seeds as a mock control. The tray was kept in an incubator under 25/16°C day/night temperatures and a 12 h photoperiod for 12d.

Supplementary Figure 3 Histopathological images of infectious hyphae in the inner epidermis of wheat coleoptiles inoculated with Δ*Fpppr1* plugs 14 dpi. **(A)** Inoculation of wheat coleoptiles in a plastic tray and infectious hyphae expending in host cells without discoloration **(B)**. Scale bars indicate 20 μm.

Supplementary Table 1 Primers used in this study for gene knockout.

Supplementary Table 2 Expression profiles on oxidoreductive-related genes in Δ*Fpppr1* mutant.
