# Supplementary material for: The FpPPR1 Gene Encodes a Pentatricopeptide Repeat Protein That Is Essential for Asexual Development, Sporulation, and Pathogenesis in Fusarium pseudograminearum
Source: Front Genet. 2021 Jan 15;11:535622. doi: 10.3389/fgene.2020.535622 (PMC7874006; doi:10.3389/fgene.2020.535622)
Supplement: Supplementary file 1 [file Presentation_1.pdf]

Legend for Supplementary video：

The fungal spores from the complemented strain cFpppr1with FpPPR1-GFP fusion construct driven by its native promoter were collected and hyphae prepared following the ways described in Figure 3 and Figure 4, respectively. The samples were stained with Mito-Tracker Red CMXRos M7512, a red-fluorescent dye that stains mitochondria in live cells. The video showing a dynamic level of two signals inside the tissues were taken with a Carl Zeiss Microscope Axio Imager M2 through two channels. Yellow signal indicates co-localization of the FpPpr1-GFP green and Mitro-Tracker red fluorescence signals.

Hyphae avi-1 to Hyphae avi-3 were for the hyphae sample staining. Scale bars indicate 5 μm.

Spore avi-4 was for spore sample. Scale bars indicate 5 μm.
