# Supplementary material for: The FpPPR1 Gene Encodes a Pentatricopeptide Repeat Protein That Is Essential for Asexual Development, Sporulation, and Pathogenesis in Fusarium pseudograminearum
Source: Front Genet. 2021 Jan 15;11:535622. doi: 10.3389/fgene.2020.535622 (PMC7874006; doi:10.3389/fgene.2020.535622)
Supplement: Supplementary Table 1 — Primers used in this study for gene knockout. [file Data_Sheet_1.docx]

**Supplemental document:** The identification procedure of the disrupted genes via *Agrobacterium tumefaciens*-mediated insertion

*Fusarium oxysporum* f. sp. *niveum* (*FON*) strain FON-11-06 was also a local isolate that was isolated from watermelon by our collaborator Dr. Shen Ling at Horticulture Research Institute in Henan Academy of Agricultural Sciences, Zhengzhou, Henan of China. A monoconidial culture was grown on PDA plates for 12 d at room temperature, and then conidia were collected by filtering through three layers of lens paper (Beijing Solarbio Science & Technology Co., Ltd). For ATMT (*Agrobacterium tumefaciens*-mediated transformation). *A. tumefaciens* strain AGL1, provided by Wang Zhengyi (Zhejiang University), bearing a binary vector pATMT1 and was grown at 28°C for 2 days in LB media supplemented with kanamycin (50 μg/mL) for transformation of the FON-11-06 strain. Transformants of FON-11-06 were inoculated on cellophane-covered PDA plates for 6 d and were harvested to extract genomic DNA.

***Agrobacterium tumefaciens*-mediated transformation and genomic DNA isolation of *FON***

ATMT was used to construct the insertion mutant library for *F. oxysporum* f. sp. *niveum* (*FON*) strain FON-11-06. The binary vector pATMT1-eGFP contained a T-DNA harboring the hygromycin B resistance (*hph*) under control of the *Aspergillus nidulans* trpC promoter. The pATMT1-eGFP was derived from pATMT1 by insertion of the eGFP cassette into the *Hind* III restriction enzyme site in the multiple cloning site driven by the *A. nidulans* *GPDA* gene (encoding glyceraldehyde-3-phosphate dehydrogenase) promoter. The T-DNA containing *HPH* and eGFP were inserted into the genome of FON-11-06 and the insertion mutant would be screened for green fluorescent signal for easy tracking of the defect.

The procedure for ATMT of *FON* was as follows. Agrobacterium *tumefaciens* strain AGL1, carrying a binary vector pATMT1-eGFP, was grown at 28°C for 2 days in LB liquid media amended with kanamycin (50 μg/mL), OD_600_ = 0.6. *Agrobacterium tumefaciens* cells were diluted in induction medium (IM) (K-Buffer 10 mL (K_2_HPO_4_ 1.84 g/L, KH_2_PO_4_1.45 g/L, pH 4.9), 0.6 g MgSO_4_, 0.3 g NaCl, 0.026 g CaCl_2_, 0.5 g NH_4_NO_3_, 5 mL C_3_H_8_O_3_, 2 g C_6_H_12_O_6_, pH5.3-5.4), both in the presence (IM+AS) and absence (IM-AS) of 200 μM acetosyringone (AS), and grown to OD_600_ = 0.15 for an additional 6 h. A total of 100 μL of this culture was mixed with an equal volume of fresh conidial suspension from strain FON-11-06 (1×10^6^ conidia per mL). For ATMT, fresh conidia at 1×10^6^/mL was used. Protoplasts were used for transformation of *F. pseudograminearum.*

The mixture (200 μL per plate) was then plated on cellophane paper covering co-cultivation medium (same as IM except that it contained 5 mM glucose instead of 10 mM glucose) in the presence or absence of 200 μM AS. Following incubation at 22°C for 48 h, the filter was transferred to PDA containing hygromycin B (200 μg/mL), streptomycin (60 μg/mL), and cefotaxime (300 μg/mL) until transformants appeared. Finally, individual transformants were transferred into 24-well PDA plates supplemented with hygromycin B (200 μg/mL) for further screening and subsequent analysis. We initially transformed *F. oxysporum* f. sp. *niveum* (*FON*) a wild type strain FON-11-06, the causal agent of watermelon wilt disease, using *Agrobacterium tumefaciens* with T-DNA including *hph* and *eGFP*. A T-DNA insertion library of FON-11-06 was generated by the ATMT system. We obtained an insertion mutant, W100D5, with severe defects from screening the library and the T-DNA flanking genomic sequence from the insertion site was identified, while the whole genome sequence of *FON* was not available. We BLAST the insertion site flanking sequence against *F. oxysporum* f. sp. *lycopersic* 4287. The alignment result showed that the T-DNA insertion was 261 bp away from the start codon of a predicted gene FOXG_08514 encoding a pentatricopetptide (PPR)-containing protein. Further BLASTp against the *F. pseudograminearum* genome database using the predicted FOXG_08514 amino acid sequence identified the orthologous gene FPSE_02553.

The mycelial mat from 6-day cultures of transformants on top of the cellophane (Solarbio No. YA0620) laid on PDA, and the mixtures of conidia and *Agrobacterium* were spread evenly on the surface of cellophane paper. 48 h later, the cellophane was sliced into 0.4 cm strips and transferred onto a selective plate for another 48 h of incubation until the mold clumps emerged. Mold covering potato dextrose plates were harvested and ground to the fine powder in liquid nitrogen for genomic DNA (gDNA) extraction using a fungal genomic extraction kit (Sangon Biotech, Shanghai, China) following the manufacturer’s instructions. gDNA concentration was determined by spectrophotometry and digested by different restriction enzymes, including *Eco* RI (G/AATTC), *Sac* I (GAGCT/C), *Kpn* I (GGTAC/C), *Sma* I (CCC/GGG), *Bam* HI (G/GATCC), *Xba* I (T/CTAGA), *Sal* I (G/TCGAC), *Pst* I (CTGCA/G), and *Hind* III (A/AGCTT), respectively, in 50 μL of reactions: 5 μL (1 μg) of genomic DNA, 10 U of restriction endonuclease, 0.5 μL 100 x BSA, 5 μL 10 x buffer, and ddH_2_O up to 50 μL. After digestion for 2 h (15 min for part digestion of *Hind* III or Sac I) at 37 °C, the restriction endonucleases were subsequently heat-inactivated at 65°C for 20 min, and the digested gDNA was purified using AxyPrep PCR cleanup kit (Nucleic acid purification kit). The resulting digested DNA fragments were circularized using a T4 DNA ligation. DNA ligation reactions consisted of (50 μL): 0.3 μg of digested DNA, 50 units of ligase, 5 μL 10x buffer, and ddH_2_O up to 50 μL at 16 °C, overnight. Ligated DNA was precipitated with ethanol in the presence of 10 mg glycogen, and the DNA pellet was resuspended in 20 μL ddH_2_O. Using circularized DNA as a template, highly efficient nested inverse (HENI)-PCRs were performed with LongAmp Taq (NEB, Biolab).

HENI-PCR amplifications were carried out by two rounds of PCR with LongAmp Taq (NEB, Biolab). The first-round PCR reactions used 1 μL circularized DNA, 5 μL 5 x buffer, 1 μL 10 μM dNTP, 1 μL of 10 μM forward and reverse primers, and 1 μL LongAmp Taq brought to a final volume of 25 μL with ddH_2_O. The second round of PCR reactions were similar to the first with the nested primer sets except that 1000x diluted products of the first-round PCR products were used as templates instead of circularized DNA. The PCR cycling profile started with 94°C for 2 min following by 35 cycles of 94°C for 30 s, 56°C for 45 s, and 72°C for 5 min with the final 72°C extension for 10 min. The primers for isolating the sequences flanking the T-DNA region of interest are shown in Table S1 and Figure 1. The purified PCR product was sent to Sangon Biotech (Shanghai) for Sanger sequencing using an ABI 3730XL DNA Analyzer. The E-value for BLASTn alignments was typically less than 10^-5^. The protein assessment from local BLASTp of the genomic database of special species at http://fungi.ensembl.org/ or the general NCBI BLASTp revealed that the amino acid identity reached to 25%, indicating an ortholog to the query. The sequences have not yet been deposited in GenBank.

The products of the second round PCR were separated on a 1% agarose gel stained with Gold view (Biotium), the PCR products were purified from the gel and then were sent to Sangon Biotech Co., Ltd. (Shanghai, https://www.sangon.com/) for sequencing with ILP2 primers for unknown sequences flanking inserted T-DNA on the left and IRP4 for sequences on the right. Sequences were analyzed using DNAstar software (Bioinformatics Software for Life Science-DNASTAR, [www.dnastar.com](http://www.dnastar.com)) and BLASTn (Nucleotide BLAST on National Center for Biotechnology Information, https://www.ncbi.nlm.nih.gov/). The predicted protein was analyzed by TPRpred in the MPI Bioinformatics Toolkit (<https://toolkit.tuebingen.mpg.de/#/tools/psiblast>).

To evaluate the pathogenicity of *FON* strains, sprouting watermelon seeds were inoculated. Briefly, seeds of watermelon cultivar Kaixuan were surface-sterilized with 2% sodium hypochlorite solution for 5 min, rinsed with sterile water, and incubated at room temperature. The conidia were collected from the 10-d-old PDA plate cultures after rinsing with 20 mL sterile water, filtered by sterile lens paper, and adjusted to the concentration at 1x10^5^/mL for inoculation. Four to five germinating seeds were put onto wet sterilized matrix in a plastic tray cell, drop inoculated with 0.5 mL of *FON* spore suspension at 1 x 10^5^/mL per germinated seed, and covered with wet sterilized matrix. The sterile water was dropped onto seeds as a mock control. The tray was transferred to an incubator under 25/16℃ day/night temperature and a 12h photoperiod.

**Isolation of T-DNA flanking genomic DNA of *FON***

By screening the T-DNA insertion mutant library in *FON*, we obtained a mutant W100D5, which had significantly reduced growth. The pathogenicity test showed that the mutant W100D5 had lost virulence on watermelon seedlings, while the wild type caused death of watermelon seedlings at 12 dpi (Figure S2). To obtain the T-DNA insertion site for mutant W100D5, genomic DNA treated with *Pst* I was used as the template. Two rounds of PCR products were sequenced using the ILP2 primer for left unknown flanking sequences and a 560 bp genome sequence was recovered. The T-DNA flanking sequence was aligned to a hypothetical protein RNA FOXG_08514 in *Fusarium oxysporum* f. sp. *lycopersici* 4287. A T-DNA was inserted in the promoter region of FOXG_08514, 261 bp away from the start codon ATG (Supplemental data). FOXG_08514 was predicted to encode a pentatricopeptide repeat (PPR)-containing protein. The whole-genome sequence was not available. Local BLAST of the FOXG_08514 amino acid sequence against the genome of *F. pseudograminearum* was performed and we identified the orthologous gene **FPSE_02553.**


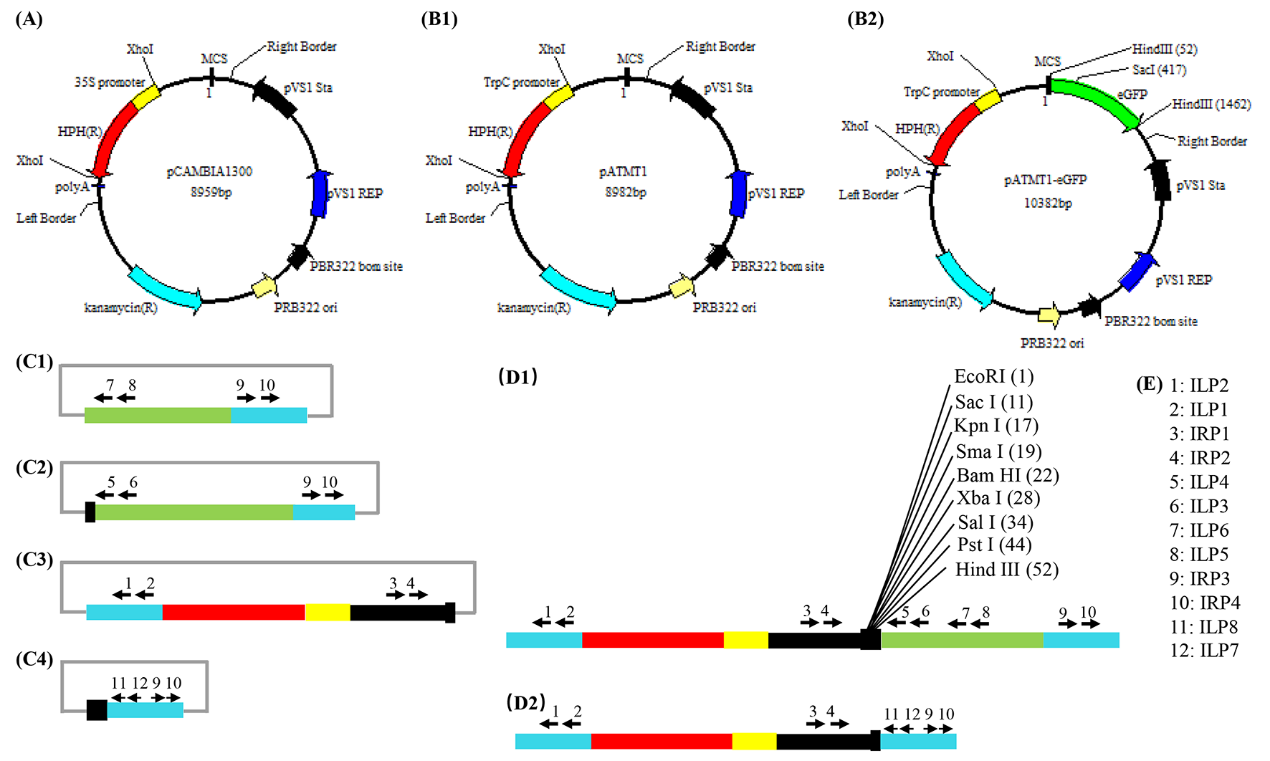


Supplementary Figure 1 | Vectors used for Agrobacterium tumefaciens-mediated transformation of Fusarium oxysporum f. sp. niveum (FON) strain FON-11-06, the special structure of the T-DNA region in pATMT1-EGFP and pATMT1 vectors for high-efficiency nested inverse (HENI)-PCR, and a schematic diagram for identifying the sequences flanking the T-DNA region of interest. (A) pCAMBIA1300 vector, (B1) pATMT1 vector, and (B2) pATMT1-EGFP vector; Panels (B1) and (B2) were suitable for HENI-PCR amplification of unknown sequences flanking both borders of T-DNA in transgenic FON mutants; (C1–C3) schematic diagram for identifying sequences flanking the T-DNA region of interest; (D) special T-DNA structure for HENI-PCR in pATMT1-EGFP vector and primer design. (E) Numbers 1 to 12 in panel (C) correspond to 12 primers, namely ILP, ILP1, IRP1, IRP2, ILP4, ILP3, ILP6, ILP5, IRP3, IRP4, ILP8, and ILP7, respectively.


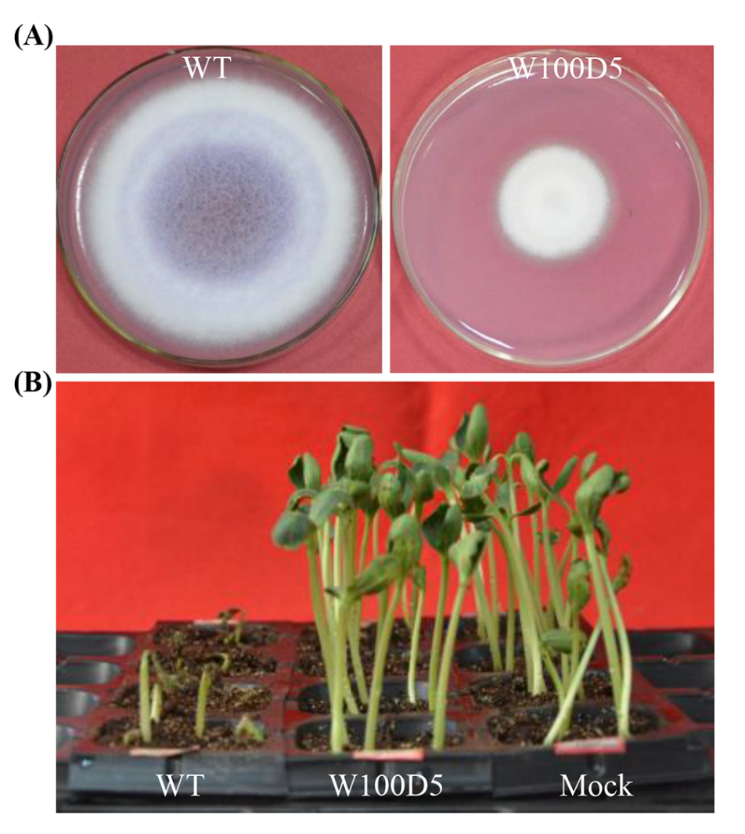


Supplementary Figure 2 | Morphology and pathogenicity assay for the mutant W1005D in FON strain FON-11-06. (A) The wild type and mutant cultures on PDA plates after 7d at room temperature. (B) The sprouting watermelon seeds were inoculated directly with 0.5 mL of the spore suspension at 1 × 105/mL in the tray cells. The sterile water was dropped onto seeds as a mock control. The tray was kept in an incubator under 25/16℃ day/night temperatures and a 12 h photoperiod for 12d.


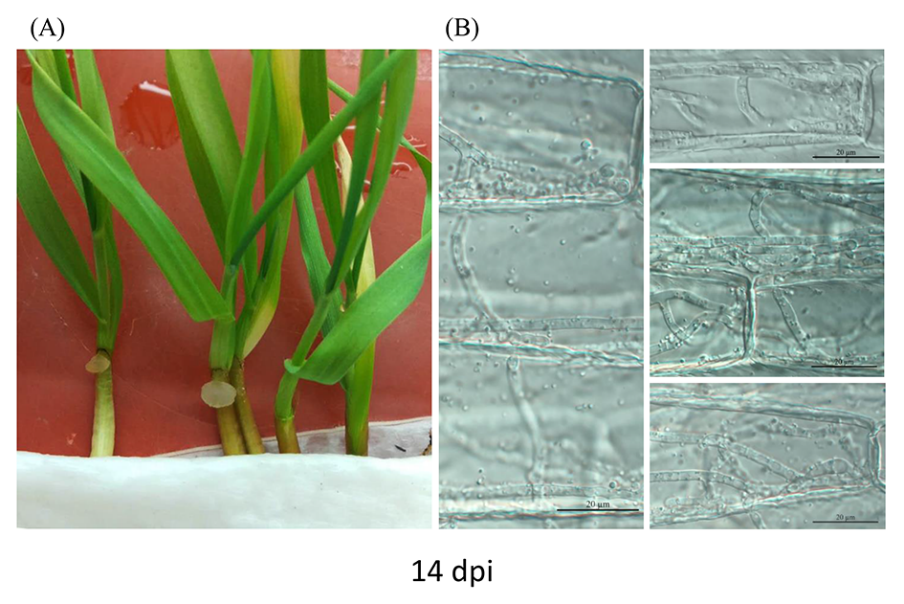


Supplementary Figure 3 | Histopathological images of infectious hyphae in the inner epidermis of wheat coleoptiles inoculated with ∆Fpppr1 plugs 14 dpi. (A) Inoculation of wheat coleoptiles in a plastic tray and infectious hyphae expending in host cells without discoloration (B). Scale bars indicate 20 μm.

Table S1 Primers used in this study for gene knockout

| **primers** | **Sequence(5'-3')** | **Description** |
| --- | --- | --- |
| GFP-F | GCAAGCTTGAATTGGGTACTCAAATTGGTTC | GFP gene detection |
| GFP-R | GGAAGCTTATCATCATGCAACATGCATGTAC |  |
| FpPPR1-F1 | GGTTGACGAAGCCAAGTCGGATGC | for upstream fragment |
| FpPPR1-R1 | TTGACCTCCACTAGCTCCAGCCAAGCCGCAACGAGTCCGAATTAGGCTG |  |
| FpPPR1-F2 | ATAGAGTAGATGCCGACCGCGGGTTCATGGCTCAACTACTCTGCAATCTTG | for downstream fragment |
| FpPPR1-R2 | ACACTGATGACCGTCCTATTCTAAG |  |
| FpPPR1-NF | GAGGAAGAGGACGCAACAGA | negative PCR |
| FpPPR1-NR | GCTCAACCTTCTATTACTCGTCAG |  |
| FpPPR1-PF | CCTACACAGAGCCATCTACTACCATG | positive PCR |
| FpPPR1-PR | GCTTTCCAACCCAGATGATGA |  |
| HYG/F | GGCTTGGCTGGAGCTAGTGGAGGTCAA | hygromycin gene detection |
| HY/R | GTATTGACCGATTCCTTGCGGTCCGAA |  |
| YG/F | GATGTAGGAGGGCGTGGATATGTCCT |  |
| HYG/R | GAACCCGCGGTCGGCATCTACTCTAT |  |
| H856F | GTCGATGCGACGCAATCGT | positive PCR |
| H855R | GCAACTGGTCAGATCAGC |  |
| FpPPR1-GFP-F | TGGGTACCGGGCCCCCCCTCGAGCCTACACAGAGCCATCTACT | for complementary fragment |
| FpPPR1-GFP-R | CTTATCGATACCGTCGACGTTATCATAAGTGTTCCGTCC |  |

Supplementary Table 1 | Primers used in this study for gene knockout.

Table S2 Expression profiles on oxidoreductive-related genes in Δ*Fpppr1* mutant

| **Geneid** | **logFC** | **pfam** |
| --- | --- | --- |
| FPSE_08050 | -7.03392 | PF00175,Oxidoreductase NAD-binding domain;PF00258,lavodoxin;PF00067,Cytochrome P450;PF00667,AD binding domain |
| FPSE_08176 | -4.68219 | PF13434,L-lysine 6-monooxygenase (NADPH-requiring);PF07992,Pyridine nucleotide-disulphide oxidoreductase;PF00743,lavin-binding monooxygenase-like;PF13450,NAD(P)-binding Rossmann-like domain |
| FPSE_02237 | -3.72397 | PF01408,Oxidoreductase family, NAD-binding Rossmann fold |
| FPSE_00389 | -2.75854 | PF13450,NAD(P)-binding Rossmann-like domain;PF00890,AD binding domain;PF01494,AD binding domain |
| FPSE_05514 | -2.67491 | PF13738,Pyridine nucleotide-disulphide oxidoreductase;PF00070,Pyridine nucleotide-disulphide oxidoreductase;PF13434,L-lysine 6-monooxygenase (NADPH-requiring);PF07992,Pyridine nucleotide-disulphide oxidoreductase |
| FPSE_05576 | -2.29233 | PF05368,NmrA-like family;PF01370,NAD dependent epimerase/dehydratase family |
| FPSE_08474 | -2.28191 | PF00724,NADH:flavin oxidoreductase / NADH oxidase family |
| FPSE_05266 | -2.18616 | PF02737,3-hydroxyacyl-CoA dehydrogenase, NAD binding domain;PF00725,3-hydroxyacyl-CoA dehydrogenase, C-terminal domain |
| FPSE_06206 | -1.97132 | PF05221,S-adenosyl-L-homocysteine hydrolase;PF00670,S-adenosyl-L-homocysteine hydrolase, NAD binding domain;PF02826,-isomer specific 2-hydroxyacid dehydrogenase, NAD binding domain |
| FPSE_04661 | -1.84124 | PF00175,Oxidoreductase NAD-binding domain;PF17147,Pyruvate:ferredoxin oxidoreductase core domain II;PF01558,Pyruvate ferredoxin/flavodoxin oxidoreductase;PF00667, AD binding domain |

Supplementary Table 2 | Expression profiles on oxidoreductive-related genes in *∆Fpppr1* mutan.
